# A clinical activation protocol improves response time to urgent/emergent neurosurgical care in children with brain tumors and intracranial hemorrhage

**DOI:** 10.1007/s00381-026-07436-0

**Published:** 2026-08-26

**Authors:** Rishi Jain, Hinda Najem, Shreya Mukherjee, Megan Votoupal, Kaethe Leonard, Julia Martorana, Yiannis Katsogridakis, Michael DeCuypere

**Affiliations:** 1 Division of Pediatric Neurosurgery, Ann & Robert H. Lurie Children’s Hospital of Chicago, Department of Neurological Surgery, Northwestern University, Chicago, 225 East Chicago Avenue, Box 28, IL USA; 2 Center for Quality and Safety, Ann & Robert H. Lurie Children’s Hospital of Chicago, Chicago, IL USA; 3 Division of Emergency Medicine, Ann & Robert H. Lurie Children’s Hospital of Chicago, Department of Pediatrics, Northwestern University, Chicago, IL USA

**Keywords:** Intracranial emergencies, Non-traumatic intracranial hemorrhage, Intracranial tumor, Pediatric patients, Multidisciplinary workflow, Time to response

## Abstract

**Introduction:**

While there are well-established protocols for trauma and ischemic stroke care, there are few dedicated to brain tumors or non-traumatic intracranial hemorrhage (ICH), especially within pediatric neurosurgery. At Ann & Robert H. Lurie Children’s Hospital of Chicago, a neurosurgical emergency activation protocol for non-traumatic intracranial emergencies, Code Intracranial Emergency (CIE), was developed and applied in 2021. This is a multidisciplinary protocol with synchronized workflows, predetermined roles, scripted communication, and streamlined patient assessment. Here we examine the effectiveness of this protocol in time management and access to care at a large tertiary referral center.

**Methods:**

Sixty-four patients with either a brain tumor or a non-traumatic ICH were retrospectively reviewed. We utilized a case–control study design for comparison purposes (January 2016–August 2022 [no code deployed, control cases, *n* = 36]; September 2021–January 2023 [CIE deployed, *n* = 28]). Both cohorts were matched for age and diagnosis/pathology. Group comparisons were conducted across time intervals (time from the emergency department (ED) to initial imaging, time to initial neurosurgery consultation, time to operating room (OR) arrival, and total length of hospital stay).

**Results:**

Across all tumor patients, the protocol significantly reduced the time from consultation to OR arrival (*p* = 0.03). For urgent/emergent tumor-related cases, significant improvements were observed in consultation to OR arrival time (*p* < 0.01). In addition, protocol utilization led to a significant reduction in consultation to OR time (*p* = 0.024) and lowered average ED to initial imaging time (56 min vs. 1.19 h) for urgent ICH cases.

**Conclusions:**

Implementation of CIE effectively reduced response time to OR arrival for patients suffering from intracranial tumor and ICH emergencies. While this cohort is underpowered, this analysis highlights the importance of a multidisciplinary protocol for optimal workflow during neurosurgical emergencies, where timely and streamlined communication is crucial for effective access to care.

## Introduction

Pediatric intracranial emergencies carry devastating consequences for patients if not addressed swiftly. Stroke in children ranks among the top causes of pediatric mortality, with nearly half categorized as hemorrhagic [1, 2]. Hemorrhagic and ischemic strokes in pediatric populations are associated with poor prognosis [3], with one in six children dying after non-traumatic intracranial hemorrhage (ICH), and most survivors experiencing persistent, debilitating long-term neurological deficits [4]. Likewise, intracranial tumors are a leading cause of morbidity and mortality among children and can clinically present in acute emergency settings as rapidly increasing intracranial pressure with potential for cerebral herniation [5, 6]. Urgent intervention, including placement of an external ventricular drain (EVD) or surgical evacuation/resection, is the most effective emergent method of management [7]. Rapid response and access to care is thus a critical pillar of addressing such emergencies for pediatric patients.

The emergency department (ED) often serves as the initial site of contact and the primary location for diagnosis, clinical stabilization, and treatment planning. It is imperative that the ED team promptly recognize signs of intracranial emergencies and simultaneously communicate with the neurosurgical and critical care teams with minimal delay [8]. Multidisciplinary collaboration between emergency medicine physicians, radiologists, critical care physicians, neurosurgeons, and patient transport teams alike must be coordinated. Tight communication and structured workflow between several teams from the ED to the operating room (OR) can substantially optimize chances of a favorable outcome [9, 10]. The importance of time in acute neurologic care for adults is well-established, and several interventions are guided by standardized benchmarks. For instance, “door-to-needle” time for administering thrombolysis in acute ischemic stroke is recommended to be less than 60 min following patient arrival [11]. Building on these guidelines, groups have proposed rapid response protocols for traumatic brain injury (TBI) with demonstrated survival benefits [12, 13]. However, unlike adult stroke or trauma frameworks, there are no consensus-driven time targets for acute management of pediatric non-traumatic ICH or brain tumor emergencies. The pediatric emergency literature exhibits broad concepts and significant variability in care delivery [14]. Even in the context of pediatric arterial ischemic stroke, a relatively well-characterized non-traumatic pediatric emergency, the National Institute of Health (NIH)-funded Thrombolysis in Pediatric Stroke trial noted multiple barriers [15], including delayed stroke recognition, imaging bottlenecks, and fragmented coordination between multidisciplinary care team members due to the lack of a pre-established acute response infrastructure. It is evident that pediatric neurocritical care requires dedicated frameworks well-tailored to its unique population and demands.

**Fig. 1:**
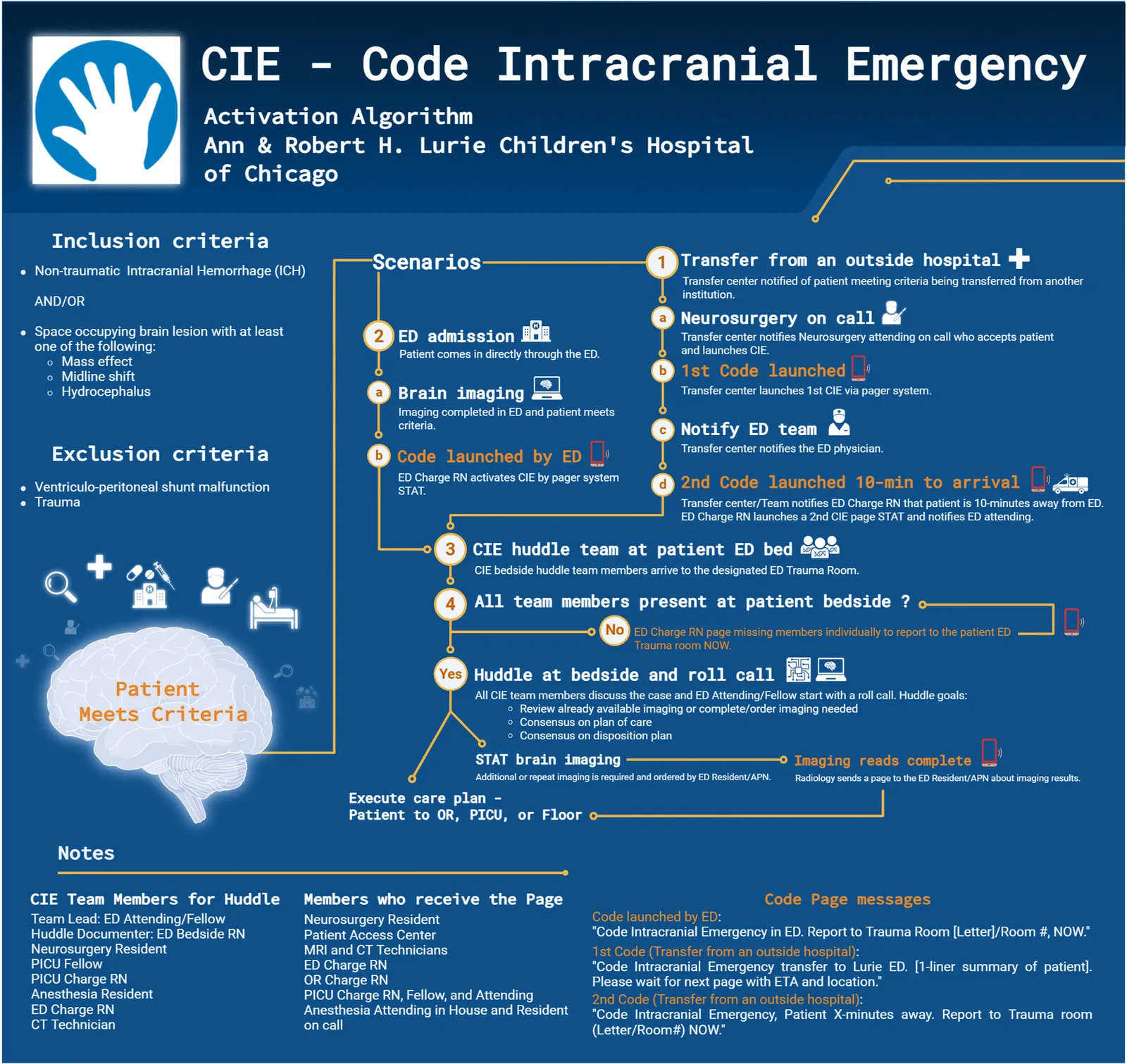
Code Intracranial Emergencies Activation Algorithm at Lurie Children’s Hospital of Chicago denoting step by step process of protocol activation in two scenarios where the patient meets criteria and is either admitted through the emergency department or is being transferred from an outside hospital. Created in BioRender. Votoupal, M. (2026)https://BioRender.com/flgu5wc

An emergency response protocol, Code Intracranial Emergency (CIE), was developed, simulated, and then implemented in 2021 at Ann & Robert H. Lurie Children’s Hospital of Chicago. CIE is a multi-staged protocol that was designed by key stakeholders in neurosurgery, emergency medicine, neurology, patient transport, medical imaging, transfer call center, information management, nursing, radiology, critical care, and anesthesia to streamline multidisciplinary communication and care coordination for pediatric patients with non-traumatic ICH or intracranial mass lesions/tumors (Fig. 1). Under CIE, when a case is identified in an emergency setting, an immediate electronic pager activation alerts neurosurgery, emergency medicine, critical care, radiology, and anesthesia teams concurrently. Downstream decision-making is driven by input from all teams and emphasizes timely progression of medical interventions with clear role delegation. The implementation of this protocol was assessed in the context of key time intervals, from ED admission to initial imaging, and to surgery, while assessing its impact on clinical outcomes by way of comparison against pathology-matched pre-CIE control cases. By evaluating this pilot program, we further aim to highlight best practices and offer evidence-based recommendations for time-critical response systems in pediatric care.

## Methods

### Patient population

This study was conducted at and approved by the Ann & Robert H. Lurie Children’s Institutional Review Board (IRB# 2024–6774). 64 pediatric patients were retrospectively reviewed at Lurie Children’s Hospital of Chicago (LCH) using institutional electronic health records. All patients were either part of a pre-CIE control group (January 1, 2016–August 1, 2021; *n* = 36) or CIE group (September 1, 2021–January 1, 2023; *n* = 28). Patients were included if they presented with either non-traumatic ICH or an intracranial tumor/mass lesion. Any patients with traumatic etiologies were excluded.

### Data collection and filtering

Cases were classified based on the urgency of clinical presentation and need for neurosurgical intervention. At LCH, surgical priority was defined as elective (i.e., non-urgent category; these cases were suspected of being urgent initially; however, based on the MRI findings and clinical presentation, these patients did not undergo definitive neurosurgical intervention within the first 24 h of presentation), and urgent (i.e., urgent/emergent; patients requiring definitive neurosurgical intervention within the first 24 h of ED admission based on MRI findings and/or clinical presentation, regardless of the use/need for medical management). Control and CIE patients were identified from institutional records and categorized into one of two diagnostic groups: intracranial tumor/mass lesion or non-traumatic ICH. All cases were required to have non-traumatic etiologies, and controls were matched to the CIE group based on at least 2 criteria (i.e., age and final diagnosis) (Table 1).

**Table 1:** Patients demographics in control and CIE groups

	All	CIE	Control
*Non-traumatic ICH*			
Patients	20	8	12
Median age (range), years	11.5 (0–15)	10.5 (0–14)	11.5 (0–15)
Female, *n* (%)	8 (40%)	3 (38%)	5 (42%)
*Intracranial tumor*			
Patients	44	20	24
Median age (range), years	9 (2–20)	9 (3–20)	8.5 (2–17)
Female, *n* (%)	24 (55%)	11 (55%)	13 (54%)

*CIE* code intracranial emergencies, *ICH* intracranial hemorrhage

Tumor patients (total *n* = 44; *n* = 4 elective and *n* = 40 urgent) were identified using an internal institutional tracking database. Eligible patients had a diagnosis of intracranial (infratentorial or supratentorial) tumor or mass lesion [Pilocytic/pilomyxoid astrocytoma (16/40); medulloblastoma (5/40); ganglioglioma (3/40); high-grade gliomas (8/40; diffuse midline glioma, glioblastoma, anaplastic astrocytoma); ependymoma (3/40); choroid plexus papilloma (1/40); astroblastoma (1/40); hemangioblastoma (1/40); and others]. ICH patients (total *n* = 20; *n* = 1 elective and *n* = 19 urgent) were identified as patients who presented to the ED with a non-traumatic intracranial hemorrhage [Arteriovenous malformation rupture (5/20); intraventricular hemorrhage (5/20); intraparenchymal hemorrhage (4/20); subdural hematoma (2/20); subarachnoid hemorrhage (2/20); aneurysm rupture (1/20); epidural hematoma (1/20)], defined by one or more of the following ICD-10 codes: S06.4XAA, I62.03, I61.5, I61.9, I62.00, or P52.3. All patient categorizations were validated by the senior author (MD) (Table 1).

The primary aim of the protocol is to ameliorate difficulties in interdepartmental communication and to increase time efficiency from ED arrival to imaging and/or surgical intervention at LCH. Thus, data variables included timestamps for ED arrival, initial neuroimaging, neurosurgery team notification and consultation, OR arrival time, and total hospital length of stay (LOS) (Tables 2 and 3). Outcomes, including return to OR, post-operative complications including infection and death, and unanticipated neurological deficits were further recorded to gauge patient trajectory (Table 4).

**Table 2:** Time intervals for all ICH and intracranial tumor patients

Variable	Control	CIE	*p*	*n*	Mean Time		Time SD	
					CIE	Control	CIE	Control
***Non-traumatic ICH***								
**ED to initial imaging (min)**	6	5	0.93	11	82.60	71.33	75.97	50.75
**Neurosurgery consult to OR (min)**	12	7 ^+^	0.07	19	378.71	713.25	473.85	418.62
**ED to OR (min)**	12	8	0.95	20	373.75	259.83	483.157	187.08
**Days in hospital**	12	8	0.64	20	26.63	19.42	22.08	11.35
***Intracranial tumor***								
**ED to initial imaging (min)**	18	14	0.05	32	196.36	326.83	182.93	248.12
**Neurosurgery consult to OR (min)**	24	20	*0.03	44	502.15	745.75	390.34	315.21
**ED to OR (min)**	24	20	0.06	44	505.85	754.50	414.06	445.23
**Days in hospital**	24	20	0.49	44	12.40	13.38	14.34	13.85

ED to Initial Imaging times were not available for all patients including some patients that received initial imaging at an outside hospital prior to transfer to or arrival at the Lurie Children’s Hospital emergency department

^+^One patient did not have timepoint data for Neurosurgery Consult to OR in the ICH CIE cohort due to non-traditional entry into the CIE workflow

*OR* operating room, *CIE* code intracranial emergencies, *ED* emergency department, *SD* standard deviation, *min* minute

*p-value < 0.05

**Table 3:** Time intervals for urgent-defined ICH and intracranial tumor patients

Variable	Control	CIE	*p*	*n*	Mean Time		Time SD	
					CIE	Control	CIE	Control
***Non-traumatic ICH***								
**ED to initial imaging (min)**	6	4	0.48	10	56.5	71.33	56.15	50.75
**Neurosurgery consult to OR (min)**	12	6 ^+^	*0.02	18	254.67	713.25	319.35	418.62
**ED to OR (min)**	12	7	0.69	19	263.85	259.83	356.04	187.08
**Days in hospital**	12	7	0.92	19	24.86	19.42	23.23	11.35
***Intracranial tumor***								
**ED to initial imaging (min)**	16	12	0.13	28	207.17	303.19	196.56	253.47
**Neurosurgery consult to OR (min)**	22	18	** < 0.01	40	432.94	753.86	345.13	328.33
**ED to OR (min)**	22	18	0.05	40	450.11	735.50	398.03	452.44
**Days in hospital**	22	18	0.56	40	13.28	14.14	14.88	14.21

ED to Initial Imaging times were not available for all patients including some patients that received initial imaging at an outside hospital prior to transfer to or arrival at the Lurie Children’s Hospital emergency department. ^+^One patient did not have timepoint data for Neurosurgery Consult to OR in the ICH CIE cohort due to non-traditional entry into the CIE workflow

*OR* operating room, *CIE* code intracranial emergencies, *ED* emergency department, *SD* standard deviation, *min* minute

*p-value < 0.05; **p-value < 0.01

**Table 4:** Post-operative complications and events for ICH and intracranial tumor groups

Group	*n*	Median follow-up (range), months	30-day return to OR ^+^	30-day infection, *n* (%)	90-day complications, *n* (%)	Deaths	90-day neurological deficits, *n* (%)
	***Non-traumatic ICH***						
**CIE**	8	12 (0–22)	1 (13%)	0	0	1 (13%)	3 (38%) ^a^
**Control**	12	38 (0–79)	4 (33%)	1 (8%)	0	1 (8%)	4 (33%) ^b^
	***Intracranial Tumor***						
**CIE**	20	24 (3–36)	1 (5%)	0	0	1 (5%)	7 (35%) ^c^
**Control**	24	34.5 (0–97)	1 (4%)	1 (4%)	2 (8%) ^	0 (0%)	6 (25%) ^d^

^+^30-day return to OR for EVD/shunt placement or revision

^90-day complications other than infection, i.e., hydrocephalus and ventricle dilation (*n* = 1), and pulmonary edema and respiratory distress (*n* = 1)

^a-d^ Breakdown of post-operative neurological deficits per symptom

^a^ Seizures (*n* = 2), seizures complicated by hemorrhagic event (*n* = 1)

^b^ Headache (*n* = 1), short-term memory loss (*n* = 1), vision changes (*n* = 2)

^c^ Ataxic gait (*n* = 1), limb weakness (*n* = 1), hemiparesis and proprioception deficits (*n* = 2), vision changes (*n* = 2), dysarthria (*n* = 2), dysphagia (*n* = 1), short-term memory loss (*n* = 1)

^d^ Gait imbalance and ataxia (*n* = 2), facial nerve palsy (*n* = 1), dysarthria and aphasia (*n* = 4), hemiparesis (*n* = 1), seizures (*n* = 1), limb weakness (*n* = 1)

*CIE* code intracranial emergencies, *OR* operating room, *ICH* intracranial hemorrhage

### Statistical analysis

Subgroup analyses were performed for both groups. Statistical comparisons between the two groups were performed using the two-sided Wilcoxon rank-sum test. To control for false discoveries due to multiple testing, *p*-values were adjusted using the Benjamini–Hochberg procedure. Statistical significance for all analyses was defined as a *p*-value < 0.05. All statistical analyses were conducted in R (version 4.5.0) and GraphPad Prism (version 10.4.0). Due to the retrospective nature of the study, complete data was not available for all patients at all time points, and this is noted accordingly. Each outcome-specific comparison was performed on the subset of patients for whom that variable was available.

## Results

### CIE protocol development and implementation

This protocol was initiated and developed based on the need to differentiate and expediate newly diagnosed patients suffering from obstructive hydrocephalus or mass effect due to brain tumor or intracranial hemorrhage versus established patients with hydrocephalus/ventricular shunts. A multidisciplinary team, [led by neurosurgery (i.e., the corresponding author, MD) and including the ED, imaging, and anesthesia teams, the call center and transport staff], was involved from initiation to implementation. Multiple protocol iterations were simulated during a 4-month period, and a final majority consensus was made on the final version of the protocol (Fig. 1). All concerns and changes that occurred were implemented and discussed through iterative meetings and ultimate consensus from all members.

### CIE activation

CIE is activated for patients with non-traumatic ICH (*n* = 20, with *n* = 8 in the CIE group) or with a tumor/space-occupying brain lesion (*n* = 44, with *n* = 20 in the CIE group), causing mass effect, midline shift, and/or hydrocephalus, confirmed on the MRI images and in need of neurosurgical evaluation (Fig. 1). CIE is activated in one of two ways (Fig. 1): (1) the patient transfer call center receives communication of a patient being transferred from an outside hospital (OSH). Imaging results are shared with the neurosurgeon on call who approves the transfer and CIE is initiated by the members of the transfer center; or (2) a patient presents directly to the ED and CIE is initiated after imaging workup indicates that they meet CIE criteria.

The huddle team meeting (Fig. 1 step 4) involves clinical exam and imaging review, the need for additional imaging to be completed, final diagnosis, and consensus over the plan of care and disposition to the OR vs ICU vs surgical floor.

### CIE improves time to OR intervention for urgent cases of non-traumatic ICH

**Fig. 2:**
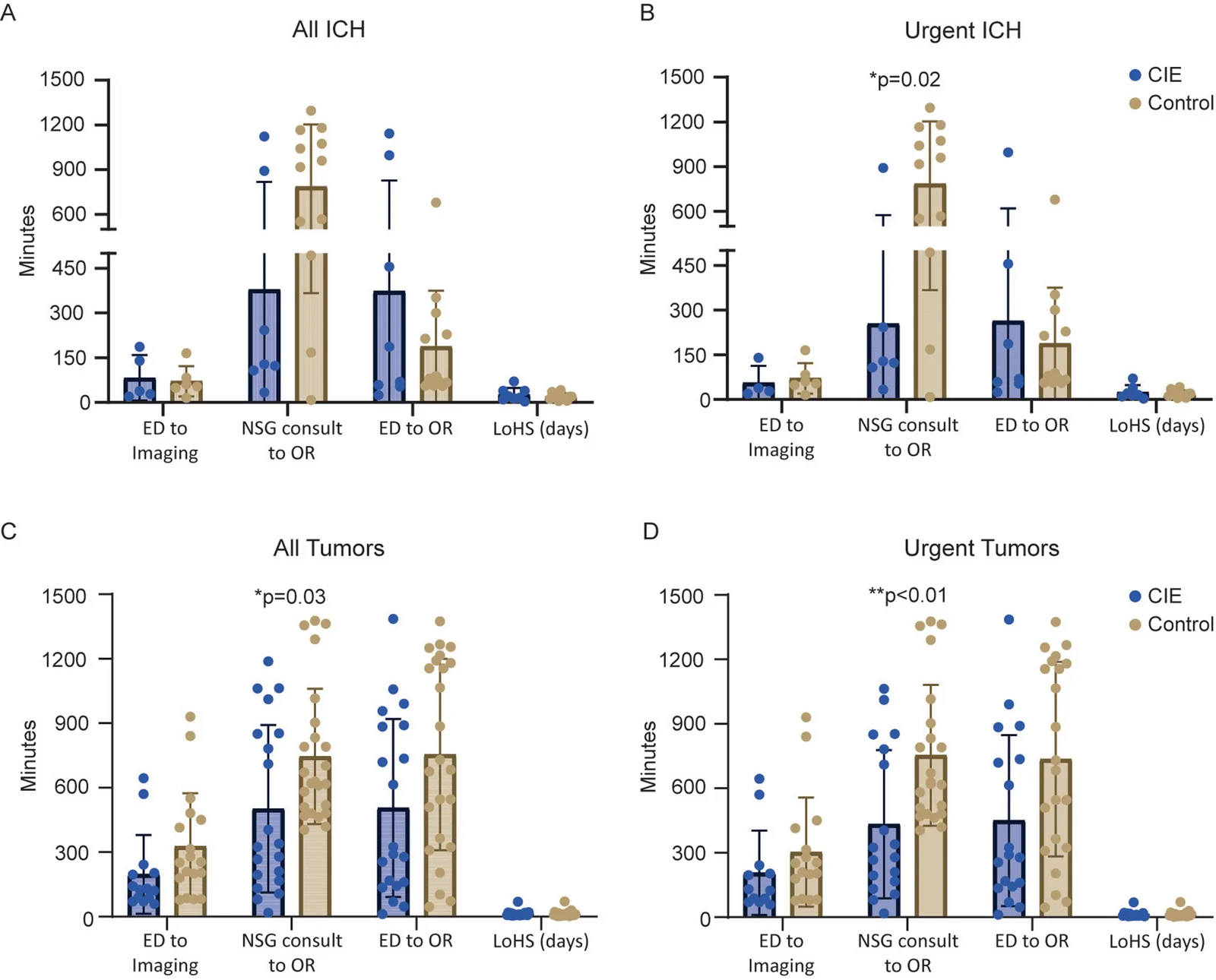
Scattered column dot plots comparing time-based outcomes between CIE and control groups for ICH and intracranial tumor patients. **A** Plots showing results for all ICH patients. **B** Plots showing results for urgent-defined ICH patients. **C** Plots showing results for all tumor patients. **D** Plots showing results for urgent-defined tumor patients. *p-value < 0.05; **p-value < 0.01

Among all ICH patients for all categories of surgical urgency, there was no significant difference in time from the ED arrival to initial imaging between CIE and control patients (*n* = 5, *n* = 6; median 38.00 vs. 57.50 min, respectively, *p* = 0.93) (Fig. 2A, Table 2). Similarly, there was no significant difference in the time between arrival to the ED and arrival to the OR between CIE and control patients (*n* = 8 and *n* = 12; median 128.5 vs 85.00 min, respectively, *p* = 0.95) and in the time between neurosurgery team consultation to OR arrival, even though CIE patients did have a lower median time to OR arrival in comparison to controls (*n* = 7 and *n* = 12; median 128.0 vs. 743.0 min, respectively, *p* = 0.07) (Fig. 2A, Table 2).

One elective case was observed in the CIE group of this cohort where a hemorrhage evacuation surgery was postponed to > 24 h after ED presentation due to the age of the patient (2 days old) and the stability of the neurological clinical exam. When this elective case was excluded (*n* = 1) and only urgent categories were assessed, there was no significant difference in the time from ED arrival to initial imaging (*n* = 4, *n* = 6; 33.00 vs. 57.50 min, respectively, *p* = 0.48) or in the time between arrival to the ED and arrival to the OR (*n* = 7, *n* = 12; median 70.00 vs. 85.00 min, respectively, *p* = 0.69) between CIE and control patients. However, there was a significant decrease in time from neurosurgical consultation to OR arrival between cohorts (*n* = 6, *n* = 12; median 125.5 vs. 743.0 min, respectively, *p* = 0.02) (Fig. 2B, Table 3).

### CIE decreased time to OR arrival in patients with a brain tumor

It is relevant to note that some patients in this cohort required additional imaging after arrival to the ED which automatically lengthened the ED stay. Four elective cases (*n* = 2 in both the CIE and control groups) were observed in this cohort and tumor resection surgery was conducted 24 h after ED arrival due to overall stability and the surgeon’s clinical decision-making.

Among all tumor patients, there was no significant difference in time from ED arrival to initial imaging (*n* = 14, *n* = 18; median 127.5 vs. 253.0 min, respectively, *p* = 0.05) and in time from ED arrival to OR arrival (*n* = 20, *n* = 24; median 307.5 vs. 706.5 min, respectively, *p* = 0.06) between CIE patients and controls (Fig. 2C, Table 2). Similarly, when elective cases (*n* = 4) were excluded from the analysis, there was no significant difference in ED arrival to initial imaging time (*n* = 12 and *n* = 16; median 127.5 vs. 230.5 min, respectively, *p* = 0.13), nor in the time from ED arrival to OR arrival (*n* = 18 and *n* = 22; median 284.5 vs. 706.5 min, respectively, *p* = 0.05) between groups (Fig. 2D, Table 3).

For all tumor cases (elective and urgent), a significant difference was observed in time from neurosurgery consultation to OR arrival between cohorts (n1 = 20, n2 = 24; median 331.5 vs. 624.5 min, *p* = 0.03) (Fig. 2C, Table 2). In addition, a significant difference was noted in the time from neurosurgery consultation to OR arrival (*n* = 18 and *n* = 22; median 301.5 vs. 624.5 min, *p* = 0.005) (Fig. 2D, Table 3) for tumor-related cases considered urgent in nature.

### Variable changes in hospital LOS and post-procedure outcomes in patients following CIE

In addition to workflow efficiency, hospital LOS and post-operative outcomes were evaluated. For ICH patients, LOS was similar between CIE patients and controls (all cases: median 18.5 vs. 19.5 days, respectively, *p* = 0.64; urgent cases: median 16.0 vs. 19.5 days, respectively, *p* = 0.92) (Fig. 2A, B; Tables 2 and 3). Post-operative complications were rare and comparable regardless of urgency level (CIE: 1/8 [13%] vs. control: 1/12 [8%]), consisting primarily of a single expected mortality and an isolated infection. New and unanticipated neurological deficits occurred in 3/8 (38%) CIE patients and 4/12 (33%) controls, consisting of seizures, minor visual changes, and short-term memory loss (Table 4).

For tumor patients, there was also no significant difference in the LOS between groups (all cases: median 7.0 vs. 10.5 days, *p* = 0.49; urgent cases: median 7.5 vs. 11.0 days, *p* = 0.56) (Fig. 2C, D; Tables 2 and 3). Complications within 90 days were infrequent and balanced between groups regardless of the urgency level (2/20 [10%] vs. 4/24 [16.6%]), including isolated reoperations, infection, other systemic events, and death. Unanticipated neurological deficits occurred in 7/20 (35%) CIE patients and 6/24 (25%) controls, most commonly focal and transient deficits such as gait imbalance/ataxia, minor visual changes, speech difficulties/dysarthria, and mild hemiparesis (Table 4).

## Discussion

Growing evidence suggests, especially in the adult emergent setting, that reducing the time to imaging and access to appropriate surgical intervention is critical to improved patient outcomes and relies heavily on streamlined multidisciplinary collaboration [16–21]. As such, it is important that emergency response strategies be standardized with algorithms centered on optimizing efficiency in patient triage, inter-team communication, and transportation. Particularly during neurosurgical emergencies, when the need for timely response is compounded by high-risk patient presentations [22], such workflows can alter patient trajectories towards better outcomes. However, no evidence is available to affirm the same for pediatric populations in such scenarios. CIE at Ann & Robert H. Lurie Children’s Hospital represents one strategy aligned with these goals and is the first structured protocol of its kind for non-traumatic intracranial emergencies in pediatric patients. In this study, the focus is on the protocol feasibility, and the impact it has on interdisciplinary communications and response times, whether it was imaging completion or surgery planning and arrival to OR.

Established standardized protocols streamline medical staff operations and workflow, subsequently reducing hospital stays and improving post-intervention outcomes in high-risk patients. For instance, evaluating the pre-hospital management of TBI, an organized trauma response system, which included protocols for field stabilization and patient transportation timing, was crucial in determining patient outcomes and long-term neurological sequelae [13]. In a quality improvement project conducted in a sixteen-bed ED in Boone Hospital Center in Missouri, the implementation of an overcrowding plan and paging protocol significantly decreased the median length of ED stay, average daily overcrowding, and radiology turnaround time, enabling more efficient patient triage and care [23]. Such studies and multiple other popular standardized algorithms (e.g., for stroke/ICH [17, 18, 24, 25], cardiac arrest [19, 26, 27], and myocardial infarction [28–31]) represent evidence that pre-delineated protocols are essential in strategic time-to-intervention reduction and complex care coordination. Pre-hospital and intra-hospital medical teams are key determinants of success and are especially integral in high-volume medical centers [32].

CIE incorporates intra-hospital interdisciplinary communication with appropriate task distribution between patient transport, call center, ED, radiology, critical care, neurosurgery, anesthesia, and OR teams to ensure timely response to incoming ICH and tumor pediatric emergencies. While it delineates critical door-to-OR milestones, the algorithm retains flexibility to accommodate heterogeneity in case severity and complexity. Importantly, the protocol incorporates proactive updates to the team, particularly during inter-hospital transfers. For example, when a patient’s arrival time is known, a page alerts the team of time-to-arrival and prompts a STAT structured bedside huddle that allows members to review available imaging and formulate a preliminary management plan.

The objective of the CIE protocol is to expedite the transport of eligible patients to the OR, a goal that was achieved in our cohort for both urgent intracranial tumor and hemorrhage cases. In both groups, the protocol was particularly effective in reducing time to OR once imaging had been completed, and the neurosurgery team consulted. In pediatric patients, such emergencies often present abruptly [33], making timely recognition of clinical warning signs, expedited imaging, and prompt neurosurgical consultation essential. Although our analysis did not specifically evaluate the diagnostic criteria or warning signs that define neurosurgical emergencies, it demonstrates that CIE improves interdisciplinary communication and accelerates appropriate escalation of care, thereby decreasing time to intervention.

The time from ED admission to neurosurgery consult can vary significantly depending on the clinical scenario. For instance, the time to neurosurgery consultation will be much longer for patients who have CIE activation within our own ED (where the initial workup is completed) compared to outside transfer situations where imaging is already completed, and the neurosurgical team is already notified that a patient is on the way. However, in other scenarios unpredicted delays during the transfer itself can arise, making times from neurosurgery consult to OR longer. Furthermore, if additional imaging is needed after LCH arrival (i.e., transfer scenario with limited or no available access to outside imaging), additional unpredictable situations may occur (i.e., difficulties in anesthesia set up, and moving child) and thus lengthen the time to arrival to the OR. These nuances can help explain the variability of the findings in the times from ED to OR versus neurosurgery consult to OR arrival between control and CIE groups.

Limiting back-and-forth patient transport can compensate for additional unpredicted time delays. For example, the neurosurgery team can review imaging in real time and make immediate clinical decisions to transfer a patient directly to the OR from the imaging suite, saving valuable time.

We acknowledge several key limitations to our study. The presented protocol is restricted to intra-hospital operations, where other non-implemented confounders (i.e., time spent at an outside ED, time to Lurie Children’s ED arrival, and early detection criteria of suspected ICH and tumor-related emergencies) may compromise its effectiveness. A potential future direction may be to extend and establish guidelines for outside healthcare workers and transport teams for more effective time-dependent identification and transport. This would also aid in triaging patients for improved time management and prioritization by the ED and CIE teams [34–36]. However, this represents a complex, large scale endeavor and may not be generalizable to a broad range of patient populations or care centers [37]. Other major limitations are the limited number of patients in the groups and the unaccounted-for variables occurring during transfer or imaging acquisition. These are largely unpredictable and can lengthen the time spent during a task, thus limiting the significance and scalability of our results.

CIE did not demonstrate a difference in hospital LOS or complications for ICH and tumor patients, concluding that at this stage this protocol has no effect on overall patient outcomes. Several factors can impact a patient’s post-operative hospital stay that are not accounted for in the CIE protocol. Of note, CIE is designed to predominantly facilitate multidisciplinary communication, patient transport, and ED operations. While LOS can be used as a proxy for patient outcomes, the lack of a significant difference is not tied to the performance of the protocol itself. In addition, the limited power of our subgroups may also explain the inability to detect differences in post-operative complications and outcomes. As such, an expanded implementation of CIE overtime would supplement several findings, enabling clearer delineation of what changes are attributable to the modified medical workflow.

## Conclusion

The implementation of CIE significantly reduced the time to OR arrival in urgent cases of intracranial tumor and ICH requiring surgical intervention, especially after imaging and neurosurgical consultation were completed. We credit its effectiveness primarily to removal of communication-based barriers and integration of a multidisciplinary workflow. While this study is underpowered and there was no effect on long-term patient outcomes, this analysis highlights the importance of multidisciplinary communication to prompt surgical management of these relatively common pediatric neurosurgical emergencies.

## Data Availability

All data generated in this study is available within the manuscript itself and no other data has been used.
